# Data on fatty acid profiles of green Spanish-style Gordal table olives studied by compositional analysis

**DOI:** 10.1016/j.dib.2017.11.038

**Published:** 2017-11-13

**Authors:** A. Garrido-Fernández, A. Cortés-Delgado, A. López-López

**Affiliations:** Departamento de Biotecnología de los Alimentos, Instituto de la Grasa (CSIC), Campus Universitario Pablo de Olavide, Edificio 46, Ctra. Utrera km 1, 41013 Sevilla, España

**Keywords:** Compositional data analysis, Fat extraction, Green table olive processing, Fatty acid, Chemometric

## Abstract

This article contains processed data related to the research published in “Tentative application of compositional data analysis to fatty acid profiles of green Spanish-style Gordal table olives” (Garrido-Fernández et al., 2018) [1]. It provides information on the implementation of compositional data analysis (CoDa) to the fatty acid profiles of Spanish-style Gordal table olives vs the use of conventional statistical analysis (data composition expressed in percentages). Particularly, it includes: i) the matrix of the sequential binary partition used for the balance estimation and the isometric log-ratio transformation (*ilr*) of the fatty acid profiles, ii) correlation among the diverse fatty acids expressed in percentages and their significances, iii) the *ilr* transformed values (*coordinates* in the Euclidean space) obtained following the sequential binary partition previously detailed, iv) the graphical presentation in the Simplex (ternary centred plot) of the treatments as a function of the four fatty acids with the higher log-ratio variances, and v) segregation of treatments based on Cluster Analysis.

**Specifications Table**

**Table:** 

Subject area	Chemistry
More specific subject area	Food Chemistry
Type of data	Tables, Figures, Text file
How data was acquired	Fatty acid profiles were acquired by analysis of their methyl esters (FAMES) in a Hewlett-Packard 5890 series II gas chromatograph
Data format	Raw, filtered and analysed data
Experimental factors	Processing phases of green Spanish-style Gordal table olives and fat extraction systems
Experimental features	The design consisted of 5 replicate treatments. Three processing phases (fresh, fermented, and packaged olives) plus two extraction systems (Abencor and Soxhlet)
Data source location	Seville, Spain, 37°21′36.5′′N; 5°56′18.6′′W
Data accessibility	The data are available with this article

**Value of the data**

• The data include the sequential binary partition of fatty acid profiles in CoDa and could be useful for calculating balances and the *ilr* transformation for other food compositions and interested researchers.
• The correlation among fatty acids expressed in percentages may help other researchers for finding spurious relationships.
• The information may facilitate the comparison of conventional multivariate techniques and compositional, regardless of the field, and promote international collaborations in data analysis.
• Presentation in the Simplex can be an appropriated way of graphing compositional data and treatments’ effects.

## 1 Data

**Fig. 1:**
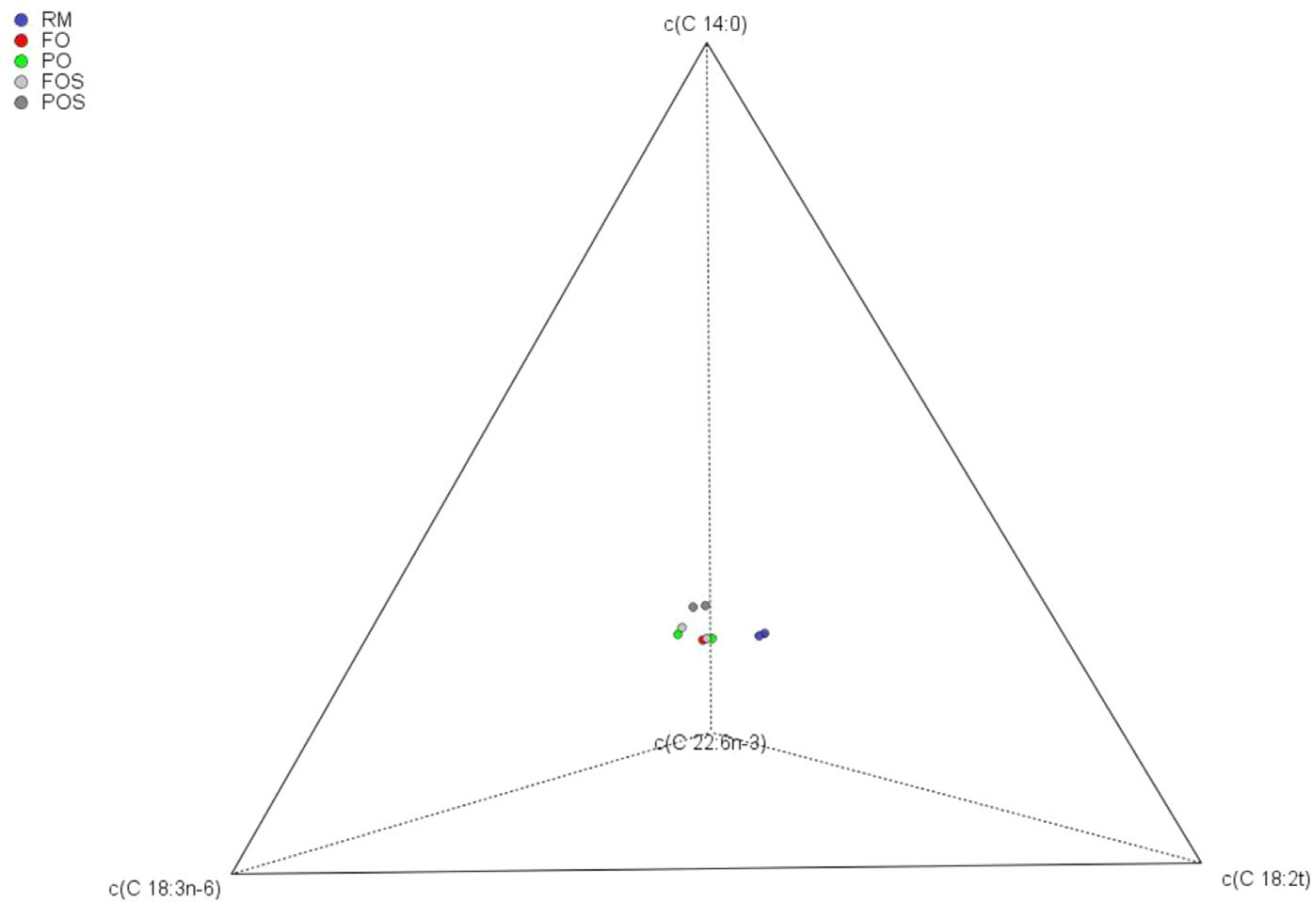
Segregation of treatments (processing phases and extraction systems), as described by a ternary centred plot based on the four fatty acids with the highest log-ratio variances. RM, oil extracted from the raw material (fresh fruits); FO and FOS, oils extracted from the fermented olives; PO and POS, oils extracted from packaged olives. S, samples extracted by Soxhlet; otherwise, by Abencor.

**Fig. 2:**
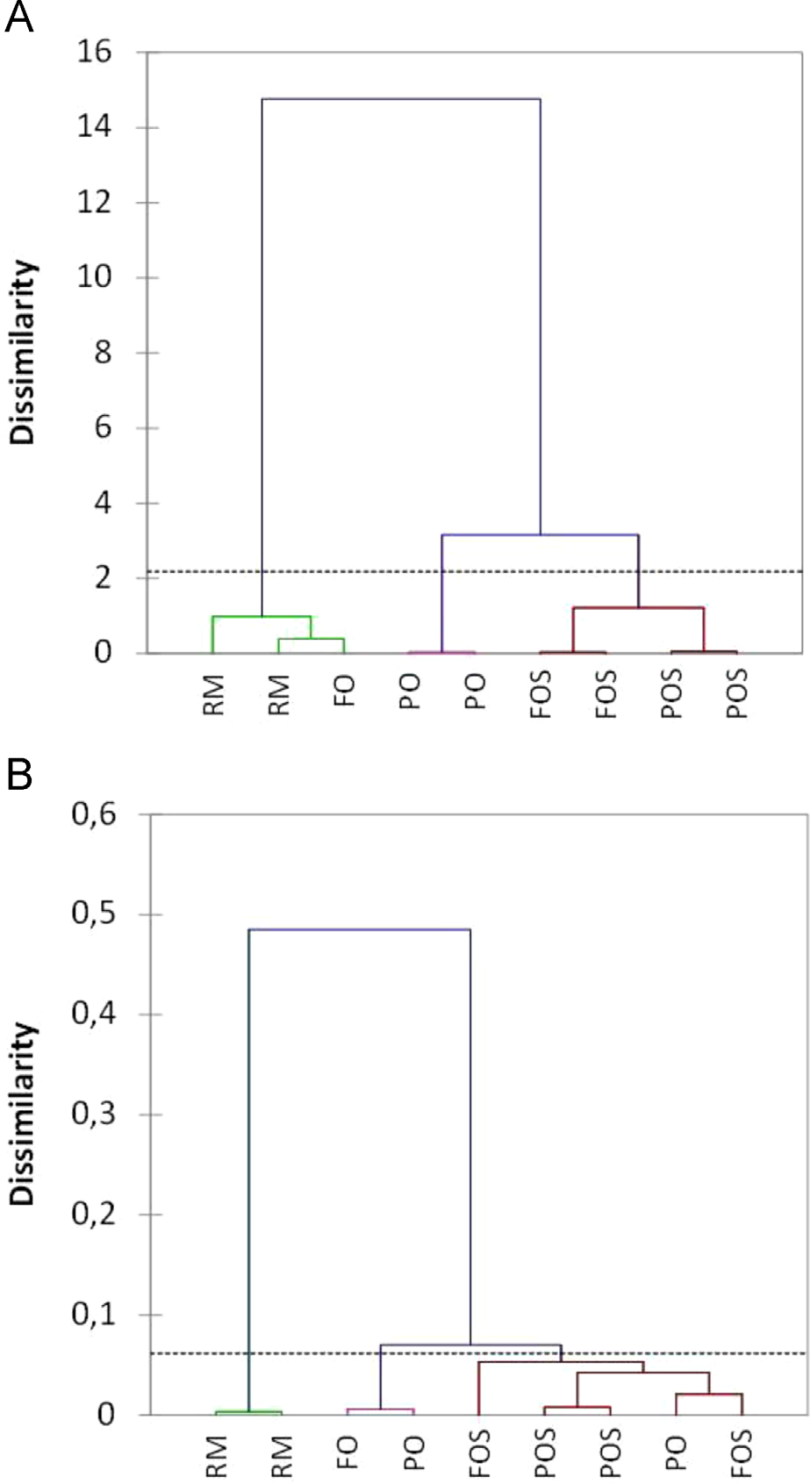
Segregation of treatments (processing phases and extraction systems), as described by Cluster Analysis, using Euclidean distance and the Ward method. A) Analysis based on percentages; B) Analysis based on *coordinates* (*ilr* transformed data). RM, oil extracted from the raw material (fresh fruits); FO and FOS, oils extracted from the fermented olives; PO and POS, oils extracted from packaged olives. S, samples extracted by Soxhlet; otherwise, by Abencor. Note that one replicate of sample FO was removed due to an apparent atypical composition.

The data cover aspects of conventional and compositional analysis. Particularly, the presentation of these data in the Simplex (Fig. 1), the binary partition (Table 1), the *ilr* transformations based on it (Table 3) as well as the application of multivariate tools to the original data (Table 2 and Fig. 2A) and *ilr* coordinates (Fig. 2B).

**Table 1:** Sequential binary partition used for balance and the *ilr* transformation calculus. In the balance, values coded as +1 are assigned to numerator; those coded −1 to the denominator; those coded as 0 do not participate in the balance. In the case of assignation of more than one fatty acid to the numerator, the denominator, or both, the balances are based on their geometric means.

Balance	C 14:0	C 15:0	C 16:0	C 17:0	C 18:0	C 20:0	C 21:0	C 22:0	C 24:0	C 15:1	C 16:1	C 17:1	C 18:1c	C 20:1	C 18:2n-6	C 18:3n-3	C 18:3n-6	C 22:6n-3	C 18:2t	Variance
1	1	−1	−1	−1	−1	−1	−1	−1	−1	−1	−1	−1	−1	−1	−1	−1	−1	−1	−1	0.0103
2	0	−1	−1	−1	−1	−1	−1	−1	−1	−1	−1	−1	−1	−1	−1	−1	1	−1	−1	0.0458
3	0	−1	−1	−1	−1	−1	−1	−1	−1	−1	−1	−1	−1	−1	−1	−1	0	1	−1	0.0043
4	0	−1	−1	−1	−1	−1	1	−1	−1	−1	−1	−1	−1	−1	−1	−1	0	0	−1	0.0014
5	0	1	1	1	1	1	0	1	1	−1	−1	−1	−1	−1	−1	−1	0	0	−1	0.0028
6	0	1	−1	−1	−1	−1	0	−1	−1	0	0	0	0	0	0	0	0	0	0	0.0003
7	0	0	1	−1	−1	−1	0	−1	−1	0	0	0	0	0	0	0	0	0	0	0.0017
8	0	0	0	1	−1	−1	0	−1	−1	0	0	0	0	0	0	0	0	0	0	0.0001
9	0	0	0	0	1	−1	0	−1	−1	0	0	0	0	0	0	0	0	0	0	0.0001
10	0	0	0	0	0	1	0	−1	−1	0	0	0	0	0	0	0	0	0	0	0.0005
11	0	0	0	0	0	0	0	1	−1	0	0	0	0	0	0	0	0	0	0	0.0011
12	0	0	0	0	0	0	0	0	0	1	1	1	1	1	−1	−1	0	0	−1	0.0010
13	0	0	0	0	0	0	0	0	0	1	−1	−1	−1	−1	0	0	0	0	0	0.0041
14	0	0	0	0	0	0	0	0	0	0	1	−1	−1	−1	0	0	0	0	0	0.0005
15	0	0	0	0	0	0	0	0	0	0	0	1	−1	−1	0	0	0	0	0	0.0002
16	0	0	0	0	0	0	0	0	0	0	0	0	1	−1	0	0	0	0	0	0.0001
17	0	0	0	0	0	0	0	0	0	0	0	0	0	0	1	−1	0	0	−1	0.0002
18	0	0	0	0	0	0	0	0	0	0	0	0	0	0	0	1	0	0	−1	0.0020

**Table 2:** Correlations among the fatty acid composition, expressed in percentages. Significant (*p*≤0.05) values are shown in bold.

Variables	C 14:0	C 15:0	C 16:0	C 17:0	C 18:0	C 20:0	C 21:0	C 22:0	C 24:0	C 15:1	C 16:1	C 17:1	C 18:1c	C 20:1	C 18:2n-6	C 18:3n-3	C 18:3n-6	C 22:6n-3
C 15:0	−0.377																	
C 16:0	0.650	0.230																
C 17:0	−0.344	**0.761**	0.067															
C 18:0	−0.435	**0.841**	0.059	**0.950**														
C 20:0	−0.220	**0.851**	0.313	**0.824**	**0.934**													
C 21:0	−0.412	**0.710**	−0.028	**0.785**	**0.885**	**0.876**												
C 22:0	−0.150	**0.724**	0.248	**0.961**	**0.867**	**0.778**	**0.678**											
C 24:0	−0.623	0.627	−0.264	**0.808**	**0.881**	**0.755**	**0.774**	0.665										
C 15:1	**0.704**	−0.590	0.289	−**0.732**	−0.647	−0.399	−0.424	−**0.687**	−**0.686**									
C 16:1	−0.544	0.020	−0.171	−0.025	0.151	0.149	0.087	−0.120	0.343	−0.188								
C 17:1	−0.285	0.542	−0.103	**0.759**	**0.717**	0.626	**0.668**	**0.791**	0.633	−0.634	0.068							
C 18:1c	−**0.719**	**0.720**	−0.135	**0.838**	**0.886**	**0.749**	**0.764**	**0.740**	**0.869**	−**0.824**	0.458	**0.701**						
C 20:1	−0.563	**0.743**	−0.078	**0.827**	**0.911**	**0.847**	**0.858**	**0.753**	**0.838**	−0.662	0.397	**0.838**	**0.932**					
C 18:2n-6	−**0.879**	0.373	−0.554	0.526	0.619	0.436	0.598	0.360	**0.783**	−**0.679**	0.659	0.576	**0.852**	**0.799**				
C 18:3n-3	0.348	−**0.812**	−0.284	−0.517	−**0.710**	−**0.841**	−**0.764**	−0.449	−0.612	0.301	−0.245	−0.347	−0.628	−**0.671**	−0.379			
C 18:3n-6	**0.687**	−0.488	0.310	−0.594	−0.474	−0.193	−0.278	−0.530	−0.455	**0.909**	−0.025	−0.361	−0.660	−0.426	−0.519	0.144		
C 22:6n-3	−0.385	−0.255	−0.582	0.218	0.065	−0.228	−0.093	0.138	0.400	−0.510	0.080	0.111	0.233	0.016	0.374	0.353	−0.478	
C 18:2t	−**0.880**	0.412	−0.603	0.605	0.623	0.360	0.531	0.406	**0.818**	−**0.784**	0.329	0.369	**0.778**	0.598	**0.831**	−0.323	−**0.747**	0.634

## 2 Experimental design, materials and methods

Olives (maturity index=1) [2] were processed in duplicate according to the green Spanish-style. After fermentation for eight months, 10 kg olives from each replicate, were packaged in glass containers (50 g NaCl/L and 5.5 g lactic acid/L cover brine), stabilized by pasteurization, and stored at room temperature (22±2 °C) for two months. The applied processing and packaging mimicked those used at industrial scale [3]. Samples ( ~5 kg olives) were withdrawn in duplicate from i) the fresh Gordal olives extracted by Abencor (RM), ii) each of the replicates of the fermented fruits (extracted by Abencor (FO) and Soxhlet (FOS)), and iii) packaged olives (extracted by Abencor (PO) and Soxhlet (POS)). The olives from the samples were pitted, homogenized with an Ultra-Turrax T25 (IKA-Labortecnik, Staufen, Deutschland) and extracted as described elsewhere [4], [5].

Fatty acid profiles were obtained through analysis of their FAMEs by GC according to the procedures recommended in the Commission Regulation (EU) No 2015/1833 [6]. The fatty acid methyl esters were quantified in a Hewlett-Packard 5890 series II gas chromatograph, using a fused silica capillary column Select FAME (100 m×0.25 mm, 0.25 μm film thickness) (Varian, Bellefonte, PA), a flame ionization detector, and a reference standard of saturated and unsaturated fatty acids methyl esters (FAME Mix C4-24). Details of the procedure can be found elsewhere [5], [7], [8], [9]. The identification of fatty acids followed the guidelines of the Commission Delegated Regulation (EU) 2015/1830 (8 July 2015) and previous works on processed olives [5], [7], [8], [9]. The analysis of each replicate was made in duplicate, and the average recorded.

The data matrix consisted of 10 rows (five treatments in duplicate) and 19 columns (fatty acids). Values were first tested for outliers and normality. The data were plotted in the Simplex (Fig. 1), analysed with specific exploratory techniques like compositional biplot [1], and subjected to sequential binary partition (Table 1). This partition led to CoDa dendrogram [1, Fig. 2] and the *ilr* transformed values (or *coordinates*) (Table 3) [10], [11]. Finally, the data (percentages and *ilr* transformed values or *coordinates*) were subjected to similar multivariate Cluster Analysis (based on the Euclidean distance and the Ward method) (Fig. 2) [12].

**Table 3:** Transformation of the compositional values into *ilr coordinates* in the Euclidean space. The sequential binary partition followed was that previously detailed in Table 1. RM. oil extracted from the raw material (fresh fruits); FO and FOS. oils extracted from the fermented olives; PO and POS. oils extracted from packaged olives. S. samples extracted by Soxhlet; otherwise. by Abencor. Notice that one replicate of sample FO was removed due to an apparent atypical composition.

Treatment	RM	RM	FO	PO	PO	FOS	FOS	POS	POS
*ilr* 1	−2.7440	−2.7413	−2.6776	−2.6723	−2.5614	−2.5991	−2.5374	−2.4716	−2.4601
*ilr* 2	−3.3817	−3.3277	−2.9100	−2.9552	−2.7339	−2.8851	−2.7701	−2.9495	−2.8814
*ilr* 3	−2.3139	−2.3043	−2.4415	−2.4213	−2.3626	−2.2157	−2.3877	−2.3961	−2.3576
*ilr* 4	−3.0939	−3.0727	−3.1012	−3.0349	−3.0607	−3.1645	−3.1350	−3.1112	−3.1259
*ilr* 5	−1.0890	−1.1001	−1.1159	−1.1510	−1.0998	−1.2378	−1.2178	−1.0831	−1.1427
*ilr* 6	−3.3214	−3.3649	−3.3166	−3.3167	−3.3614	−3.3316	-3.3174	−3.3198	−3.3384
*ilr* 7	3.5889	3.5861	3.6603	3.6446	3.6163	3.6883	3.6838	3.6765	3.7015
*ilr* 8	−0.5603	−0.5547	−0.5786	−0.5618	−0.5761	−0.5531	−0.5491	−0.5482	−0.5468
*ilr* 9	2.2300	2.2331	2.2419	2.2515	2.2266	2.2508	2.2593	2.2468	2.2396
*ilr* 10	1.1402	1.1404	1.1990	1.1971	1.1716	1.1691	1.1977	1.1921	1.1817
*ilr* 11	0.1605	0.1655	0.1446	0.1554	0.1416	0.1280	0.1874	0.2123	0.2335
*ilr* 12	0.2006	0.1992	0.2675	0.2467	0.2715	0.1894	0.2559	0.2539	0.2753
*ilr* 13	−3.7743	−3.7474	−3.6635	−3.6275	−3.6314	−3.5883	−3.5886	−3.5977	−3.6325
*ilr* 14	−0.4296	−0.4202	−0.3822	−0.4015	−0.4123	−0.3477	−0.3806	−0.3918	−0.3957
*ilr* 15	−2.1816	−2.2040	−2.2204	−2.2016	−2.1807	−2.2060	−2.1767	−2.1998	−2.1703
*ilr* 16	3.8101	3.8122	3.8106	3.8099	3.7989	3.8352	3.8073	3.8206	3.8159
*ilr* 17	3.2268	3.2263	3.2603	3.2429	3.2450	3.2081	3.2386	3.2199	3.2424
*ilr* 18	3.2520	3.2513	3.2834	3.2812	3.3005	3.3209	3.3765	3.3357	3.3744

CoDaPack v. 2.01.14 (Department of Computer Science and Applied Mathematics, University of Girona, Spain), XLSTAT 2014 (Addinsoft, Paris, France) were used for data processing and graph drawing.
