# Supplementary material for: Data on fatty acid profiles of green Spanish-style Gordal table olives studied by compositional analysis
Source: Data Brief. 2017 Nov 13;16:231–8. doi: 10.1016/j.dib.2017.11.038 (PMC5712806; doi:10.1016/j.dib.2017.11.038)
Supplement: Supplementary file 1 — Supplementary material [file mmc1.docx]

The authors of the manuscript entitled **“Data on fatty acid profiles of green Spanish-style Gordal table olives studied by compositional analysis”** declare not conflict of interest.
